# A qualitative evaluation of the specific carbohydrate diet for juvenile idiopathic arthritis based on children’s and parents’ experiences

**DOI:** 10.1186/s12969-023-00914-8

**Published:** 2023-10-19

**Authors:** Naima Hagström, Elin Lövestam, Afsaneh Koochek, Lillemor Berntson

**Affiliations:** 1 Department of Women’s and Children’s Health, Uppsala University, Uppsala, Sweden; 2 Department of Food Studies, Nutrition and Dietetics, Uppsala University, Uppsala, Sweden

**Keywords:** Arthritis, Juvenile idiopathic, Diet Therapy, Qualitative research, Specific Carbohydrate Diet

## Abstract

**Background:**

Insights into the immunological role of the gastrointestinal tract in autoimmune conditions have led to the investigation of diet as a potential adjunctive treatment option for juvenile idiopathic arthritis (JIA). The specific carbohydrate diet (SCD) has shown promising results. However, studies on participants’ experiences of dietary interventions in JIA are rare. In this study we investigated the experiences of children and parents’ who had participated in a four-week intervention with SCD aiming to examine the potential anti-inflammatory effects.

**Objectives:**

To conduct a qualitative evaluation exploring children’s and parents’ experiences of the dietary intervention, how they navigated challenges, and their support requirements.

**Methods:**

Semi-structured interviews were conducted with 12 children and 15 parents from 13 families, who were interviewed individually and together. The transcripts were analysed using systematic text condensation.

**Results:**

Most participants interviewed found the intervention beneficial, with 12 out of 13 reporting positive effects, such as reduced pain and morning stiffness, and improved gastrointestinal function. Many participants reported being willing to repeat the intervention in the current form. Despite facing challenges, all children followed the diet for one to three months, with some continuing to follow a modified version. Facing the socio-emotional consequences of adhering to the diet was challenging for children. These were handled by focusing on the positive aspects and by relying on the supportive environment available. Parents struggled with practical issues since the diet required hard work, time, and money. Areas identified as requiring additional support include finding simple, quick, and child-friendly solutions, strengthening organizational food skills such as meal planning, and preparation prior to starting the intervention regarding socio-emotional aspects.

**Conclusion:**

Navigating the dietary treatment was considered challenging, practically for the parents and socio-emotionally for the children. Based on the reported challenges and participants’ suggestions the intervention could be optimised by providing support and solutions in relation to the practical issues and better preparation regarding dealing with the socio-emotional consequences. Despite the difficulties, the participants reported overall positive experiences of, and attitudes towards, the current setup. Consequently, dietary interventions, such as the SCD, may be regarded as suitable targets for further research.

**Supplementary Information:**

The online version contains supplementary material available at 10.1186/s12969-023-00914-8.

## Background

Juvenile idiopathic arthritis (JIA) is the most common autoimmune disease in children and has an incidence of approximately 15/100,000/year in the Nordic countries [1]. The course of the disease can be unpredictable and often entails a variety of symptoms, some of the most common being persistent joint pain, fatigue, and morning stiffness, leading to a restriction in movement and activity which ultimately reduces the quality of life of those affected [2]. In addition, children with JIA have an increased risk of psychiatric morbidity [3].

While pharmacological advances have improved the long-term outcomes for children with JIA, challenges persist [4, 5]. Depending on the disease subtype and severity, treatments range from non-steroidal anti-inflammatory drugs (NSAIDs) and intra-articular cortical steroid injections to biological disease-modifying antirheumatic drugs (DMARDs); the most commonly used conventional DMARD is Methotrexate (MTX). Treatment regimens are often changed due to ineffectiveness or loss of efficacy, adverse effects, intolerance, remission or patient preference [6]. Despite this, prior research has found that an unreasonably large number of children still live with active disease [4].

The cause of JIA is unknown but environmental factors thought to alter intestinal immunity, such as early weaning and the use of antibiotics at an early age, have been shown to increase the risk of developing JIA [7, 8]. Insights into the immunological role of the gastrointestinal tract in the disease have sparked interest in diet as a potential adjunctive treatment candidate [9, 10]. However, there has been limited exploration of the use of diet as a treatment option. Currently, the specific carbohydrate diet (SCD) is the only complete dietary regimen that has been investigated as a potential adjunctive treatment for JIA [11]. In a pilot study by Berntson, the results after a four-week SCD intervention showed a significant improvement in physical function, as well as a reduction in pain and morning stiffness. The SCD has been previously studied as a treatment for inflammatory bowel disease (IBD) and primarily consists of vegetables, fruits, nuts, seeds, meat, fish, poultry, and well-fermented dairy products [12]. Notably, it excludes grains, refined sugars, and certain additives [12]. This means that most meals must be prepared completely from scratch, which requires time and effort.

Implementing and adhering to the SCD can be burdensome for both children and parents and challenges to the long-term adherence of SCD in IBD have been identified in a number of studies. The challenges include increased grocery costs, time-consuming food-related tasks, lack of taste and variety in the diet, and psychosocial impacts such as potential social isolation and friction between family members [13–15]. However, at the time of writing only one qualitative study has explored these challenges in depth. Schwartz et al. identified three barriers to SCD use in paediatric IBD: cost, time commitment, and psycho-social impact [14]. While highlighting significant barriers, the study by Schwartz only included the parental perspective, and the authors recommended that future qualitative studies also include children.

Although the SCD has yielded promising results in both JIA and IBD [11, 12], it is hypothesized that the burden of implementing and adhering to the diet may outweigh its perceived benefits, which may in turn affect compliance and effectiveness. It is, therefore, essential to investigate whether participants perceive the SCD intervention as worthwhile. Earlier findings [13–15] highlighted the need to further explore both parents’ and children’s perspectives on the feasibility, acceptability, and perceived effectiveness of SCD as an adjunctive therapeutic approach. Through qualitative trial evaluations, we can gain insights into participants’ views, which are valuable in order to identify potential issues regarding design, intervention or delivery [16]. This provides an opportunity to decide whether to proceed with further research and, in which case, to enhance the quality of the intervention before advancing to more costly and extensive trials [17]. We have therefore conducted a qualitative evaluation of the previously mentioned pilot study by Berntson.

Our purpose was to explore the experiences of children with JIA and their parents in relation to their participation in the intervention, as well as learn how they navigated the challenges and what support they required.

## Methods

### Research context

This study is a qualitative sub-study of a previous study conducted in 2017–2020 which examined the potential anti-inflammatory effects of SCD. A total of 28 children and teenagers diagnosed with JIA were included, of whom 22 completed a four-week dietary intervention [11]. The inclusion criteria were children having disease activity, although low and stable, and that both the parents and the children were strongly motivated to try the SCD. The diet was added as an adjunctive component to their existing medical treatment, which remained unchanged for 12 weeks before inclusion. Children were excluded if changes were made to their pharmacological treatment during the study. This was the case for one of the participants who needed steroid injections after three weeks into the study. Three participants found the diet too challenging and withdrew after 3, 7, and 21 days, respectively. One participant withdrew after 5 days due to a serious family-related social situation, and one participant discontinued due to adverse effects, specifically, loose stools.

Before starting the intervention, the families were given information and an introduction to the diet by a dietitian. They received written material, including a list of allowed foods and SCD-compliant products available in Swedish grocery stores. The families also received a recipe booklet produced specifically for the study, including a wide variety of recipes for all main meals, snacks and dessert options. To further assist the participants, a letter was sent to the child’s school stating that the child was participating in a research study and to request facilities for the storing and heating of food and permission for the child to bring a packed lunch from home to eat in the canteen with their friends. The letter also stated that the child was not allowed to bring foods containing nuts or almonds to school.

The families then had an optional two-week period to transition to the SCD. The first day of consumption of the complete diet was considered to be the intervention start date. The participants were asked to strictly maintain the diet for a minimum of four weeks followed by an optional, more liberal, phase consuming a modified version of the SCD where potatoes, rice and oats were reintroduced into the diet. During the trial, all participants had thorough follow-up appointments with the treating physician, access to a physician and dietitian by email, and access to the dietitian by telephone. The complete study protocol has been published elsewhere [11].

### Study design

A qualitative exploratory study design with semi-structured interviews was used. The consolidated criteria for reporting qualitative research (COREQ) guidelines were followed to ensure appropriate reporting of the results [18].

### Participants and recruitment

Participants were selected using purposive sampling [19]. Only families that had completed the four-week intervention during the preceding two years were invited to participate in order to reduce the risk of recall bias due to the time gap between inclusion in the original study and this qualitative sub-study. However, due to insufficient recruitment of participants in relation to the estimated information power [20], the inclusion criteria were later changed to include all families that had completed the four-week intervention. At the time of the interviews, some participants had become young adults; however, for simplicity, both teenagers and young adults are referred to as children in the text. The families were invited to participate by the child’s treating physician and those interested were sent further information about the study and consent forms by email. Of the 22 invited families, 13 accepted to participate, 8 were non-responders, and one declined because of difficulties remembering due to such a long time having passed since participation in the intervention.

### Data collection

Semi-structured interviews in Swedish were conducted and audio-recorded with 27 individuals from 13 families between October 2021 and March 2022. The interviews were performed in line with the Swedish Public Health Authority’s SARS-COV-2 guidelines. All interviews were held digitally in password-protected video meetings to safeguard the participants’ integrity. In all but one interview, the participants attended from their own homes. One of the interviews was held by phone due to technical problems.

Each interview lasted on average 63 min (range 38–88 min) and consisted of three parts: a shorter part with only the participating child, a longer part with both the parent(s) and the child, and a final part with only the parent(s). The purpose of separating the interviews was to enable participants to speak freely without having to censor their responses due to the other party’s presence [21]. Children were interviewed first to avoid their responses being influenced by their parent’s points of view; however, children were asked if they would rather have their parent(s) present during the interview. This was the case in one family, resulting in a two-part interview (family B). In another family, the child and mother were interviewed separately, for logistical reasons (family D). In a third family (family E), only the parent attended the interview. No repeat interviews were held and the participants were not given the opportunity to review the transcripts or the results.

The interviewer, a registered dietitian and PhD student, had prior experience of conducting semi-structured interviews with children. She had not been involved in the standard medical care of the participating children nor in delivering the intervention. However, she had previously performed interviews with five of the children for a student project in 2018 (unpublished work).

Based on the interview guide used in 2018, an extended version including open-ended questions was developed and revised after input from healthcare personnel and a patient representative (see Supplementary Table, Additional File 1). The new interview guide was pilot-tested with one family and resulted in minor changes; the pilot interview was included in the analysis. Each interview part began with general questions to gather descriptive data about the family and then continued with open-ended questions about their experience of the intervention. Probing questions were used when needed to enable the participants to provide more in-depth responses. Clinical characteristics, for example the JIA subtype according to the International League of Associations for Rheumatology (ILAR) classification [22], were extracted from data collected in the original study.

### Data processing

All data and personal information were processed in accordance with the General Data Protection Regulation. Recorded audio files were transferred to a secure server for research data storage and immediately erased from the recording device. In the collected data, the identity of each participant was concealed by labelling with a code consisting of two letters: the first was the same for all participants within a family, while the second represented gender and place within the family. The second letter could be one of four: F for father, M for mother, G for girl, and B for boy. In the text that follows the child’s age and gender are stated under each quote to enhance readability.

### Data analysis

The interviews were transcribed by the first author and a professional transcriber. Using systematic text condensation and an inductive approach, data were analysed in four steps [23]. First, an overview of the data was established by carefully reading the interview transcripts. Keeping the research question in mind, preliminary themes in the material were identified. This first step was initiated after the first three interviews had been transcribed. The analysis continued in batches of three to four transcripts in parallel with interviewing and transcription. The first three were analysed independently by the first and second authors, both trained in qualitative analysis, followed by discussion and agreement on the preliminary themes. In the second step, meaning units relevant to the research question were identified and marked with codes. The third step entailed the creation of subgroups and then systematic abstraction and condensation of the meaning units within each subgroup. A process of renaming and regrouping codes and subgroups then took place, focusing on the structural relationships between them. The quotes best reflecting the results within each final subgroup were selected and translated from Swedish into English. In the fourth and final step, the contents of the condensates were synthesised into the analytical text presented below. The second and third steps of the analysis were performed in NVivo Release 1 (Qualitative Software for Research International). For an example of the analysis, see Supplementary Table, Additional File 2.

## Results

The study sample included 13 children (of whom 12 were interviewed) and 15 parents (12 mothers and 3 fathers). Demographic data and clinical characteristics for the children are reported in Table 1. The number of family members following the SCD differed between the participating families (shown in Table 2). Most had SCD-friendly dinners where everyone could eat the main dish, with family members who were not following the diet eating optional side dishes not permitted on the SCD. None of the families reported having previously tried any special diet for JIA; however, one child followed a vegetarian diet, another ate lactose-free food, and a third suffered from nut allergy.

**Table 1:** Demographic data and clinical characteristics of participating children with Juvenile Idiopathic Arthritis (n = 13)

Variables	Median	Min - Max
Gender, girls/boys (n)	8/5	
Age at introduction of SCD (years)	11.2	8.0–17.7
Age at interview (years)	14	9–21
Duration of disease at inclusion (years)	3.3	1.5–10.8
Length of time between inclusion and interview, (years)	3.4	0.8–4.3
**ILAR Categories, course type (n (%))**		
Oligoarticular persistent	5 (38.5)	
Polyarticular RF -	4 (30.8)	
Oligoarticular extended	2 (15.4)	
Enthesis-related arthritis	1 (7.7)	
Polyarticular RF +	1 (7.7)	

SCD = Specific Carbohydrate Diet, ILAR = International League of Associations for Rheumatology, RF = Rheumatoid Factor

**Table 2:** Characteristics and description of participation and perception of the intervention

Family	Interview participants	Age of child (years)		Who else ate SCD apart from the child	Time on strict SCD (months)	Total time on SCD strict + modified ^c^ (months)	Perceived effect of the diet	Willing to redo it (parent/child)
A	M, C	18	Parents		1.5–2	24	Yes	Yes/yes
B	M, C	12	Just the child		1	6	Yes	Yes/yes
C	M, F, C	14	1 parent		1	1	Yes	No/yes
D	M, C ^a^	21	1 parent		1	> 12	Yes	Yes/yes
E	Only F	9	Parents		> 1	Ongoing ^d^	Yes	Yes/unknown
F	M, C	13	1 parent		> 1	7	Yes	Unsure/yes
G	M, F, C	11	Parents + 1 sibling		1	Ongoing ^d^	Yes	Yes/no
H	M, C	20	1 parent		> 3	> 6	Yes	Yes/yes
I	M, C	13	Parents		1	1	No	Unsure/unsure
J	M, C ^b^	13	Just the child		1	3	Yes	Yes/yes
K	M, C	17	1 parent		1	- ^e^	Yes	Yes/yes
L	M, C	17	Just the child		1	6	Yes	Yes/yes
M	M, C	14	1 parent		1	- ^e^	Yes	Yes/yes

M = Mother, F = Father, C = Child, SCD = Specific Carbohydrate Diet

^a^ Separate interview with mother and child.

^b^ Child was only interviewed with parent.

^c^ Modified SCD includes additional foods such as rice, oats and potatoes.

^d^ Occasionally consumed non-SCD foods

^e^ Participant unable to recall total time on the diet

Most interviewed participants found the intervention beneficial, with 12 out of 13 children and young adults reporting positive effects on disease symptoms after two to three weeks of adherence. Improvements included reduced pain and morning stiffness, and increased energy. Some also noticed other positive effects, such as improved gastrointestinal function and reduced hunger. The participants followed the diet for one to three months, and several continued with a modified version. The reasons for eventually returning to a regular diet were primarily social, driven by the desire to eat the same foods as others on occasions such as birthday parties, family gatherings, and especially in school. Motivation was initially high due to symptomatic improvement but waned over time as the children found themselves increasingly tempted in social situations. However, some families noted that motivation increased again when symptoms returned. In addition, the burden on the parents played a role, particularly in their decision to reintroduce more ready-made food items. Nevertheless, many reported that they continued to adhere to some of the core concepts of the diet at home while incorporating store-bought items.

In each interview, the participants were asked the same final question: if they could go back in time with the knowledge they had now after participating in the study, would they still agree to take part? They were also requested to provide a rationale for their answer. As shown in Table 2, many participants would have chosen to take part in the study again. The primary reason was improvement in the child’s symptoms.

While most families were positive to the SCD and reported multiple benefits, they also reported many challenges. The qualitative analysis resulted in two categories representing the families’ main challenges: Managing practical issues and Dealing with socio-emotional consequences (Table 3). Parents generally reported more practical issues, whereas children seemed to struggle more with socio-emotional aspects. Diverse strategies were used to navigate these challenges, as described in the subgroups: Creating the right conditions through food preparation and planning, Reducing costs, and Involving the children. Participants also identified the following areas where a need for additional support was highlighted: Simple, quick and child-friendly solutions, Strengthening organisational food skills, and Financial assistance. To deal with the socio-emotional consequences, the families chose to focus on the positive aspects and relied on the supportive and encouraging environment available. Areas where the families expressed a need for further support were preparation for how to deal with socio-emotional aspects and communication in order to preserve normalcy and mitigate feelings of isolation.

**Table 3:** Overview of the results from interviews

Challenges	Strategies/solutions	Need for support/improvement
Managing practical issues	Creating the right conditions through food preparation and planning	Strengthening organisational food skills
	Involving the children	Simple, quick and child-friendly solutions
	Reducing costs	Financial assistance
Dealing with socio-emotional consequences	Choosing to focus on the positive	Preparation regarding socio-emotional aspects and communication
	Supportive and encouraging environment	

### Managing practical issues

The main challenges for the parents revolved around the practical aspects of the dietary change. There was unanimous agreement that implementing and following the SCD required hard work, time and money. Despite having cooked regularly before the study began, planning meals, grocery shopping and preparing food proved challenging for many. When they first started the diet, they had to look for and find unfamiliar products and meticulously scrutinise every ingredient, which could mean spending hours in the grocery store. Furthermore, parents now had to make or prepare products at home which they had previously bought ready-made. Many said it felt as if they spent all their time in the kitchen, from early in the morning to late at night. Several mothers commented that it would have been ‘impossible’ to work full-time during the initial four weeks due to the considerable amount of time required in the beginning.

### Creating the right conditions through food preparation and planning

Through lots of preparation and consistent planning, adhering to the diet became easier. Over time, the families learned what products they could buy. To minimise time spent shopping, some families began ordering more groceries online or shopping in a single store and ordering specialty food products online. The parents described the importance of planning and preparing every meal, even snacks. One strategy involved preparing larger quantities and freezing the leftovers to use when there was little time to cook. A few parents spent their weekends cooking and preparing meals for the following week.

*“In purely practical terms, our solution was mainly that we, on Sundays I think, cooked a whole load of basic stuff […]. It takes a bit more time and then, during the week, you could just throw something together. So, like, preparing a lot on one day, so it doesn’t take that much time on other days. That’s one tip, I guess. So we made a lot of almond milk then, and mum maybe made chopped tomatoes, yeah, setting aside a certain amount of time, instead of having to try to do all that on weekdays.”* (Family D, girl 21 years).

Another strategy the parents used was creating an SCD-friendly environment at home. This meant eliminating food items that were not allowed by not stocking the freezer with foods they couldn’t eat, and clearing out the pantry or parts of it. One parent prepared a drawer with only SCD-friendly snacks for her child in order to help them to stay on track.

*“We started the diet by clearing out everything that we couldn’t eat. We just didn’t have it at home. So we gave stuff to neighbours and family, everything that we didn’t want anymore. And that made it easier, because I have noticed that now, if you have things at home that don’t really fit into the SCD, it can easily happen that you use them. So I think one success factor was just clearing all that out, so you only had things you could eat at home.”* (Family F, mother).

### Strengthening organisational food skills

Many parents were unaccustomed to the level of planning required to manage the new diet and were unsure where to begin. Participating parents identified their lack of organisational skills as a potential reason for not continuing the diet after the initial four weeks, since such a lot of planning and preparation was required. Those who struggled expressed a desire to be more organised. To address this, a few parents suggested that training sessions on meal planning could be offered before the start of the study. They wanted to learn how to create personalised weekly meal plans for their family, step by step, starting from a suggested meal plan.

*“I’m not the best at planning, so this thing was really hard. You couldn’t just think ‘So, what are we having?’ and take something out of the freezer or go and buy something. This required a lot more planning, and I found that difficult.”* (family L, mother).

### Involving the children

In a few families the children got involved in the kitchen to help their parents out. Preparing and cooking together at weekends became a family activity where siblings also participated. One parent recommended letting the children contribute by reading recipes and taking part in the cooking. Some children even came up with recipes for desserts and snacks. An older teen explained the benefits of taking part in the cooking process:

*“It wasn’t always the most normal food. But I think that, yeah, it was anyway tasty. I liked most of it. I often helped out when we were cooking and, then, I often feel like it’s tastier when you know what’s in it.”* (Family A, boy 18 years).

### Simple, quick and child-friendly solutions

Managing everyday life without adherence to the diet becoming too complicated was described by parents as a ‘balancing act’. In hindsight, parents questioned the amount of effort they had put into it, saying that perhaps they could have done things differently to make it simpler and less arduous. A common view was that cooking ‘shouldn’t take time; it needs to be quick’. Although the ambition of most parents was that the whole family would eat the same food, several parents cooked multiple meals to please different family members.

The recipes given out to parents elicited differing opinions. Some found them helpful as meal suggestions and said that ‘it would have been hard to manage without them’, while other families felt that suggestions for quick and ‘simpler everyday food that children and teenagers like’ were lacking. However, introducing new recipes was deemed too much of a change for some families. Many parents preferred to adapt their usual family recipes to the framework of the SCD.

*“But I think maybe simplifying what you already eat, trying to start from what the rest of the family is eating… so you don’t have to work yourself ragged.”* (Family H, mother).

### Reducing costs

Families used different strategies to reduce costs. Some limited the number of family members who ate more expensive food items. However, this often meant cooking several different versions of a meal.

*“It’s much more expensive, I mean if you go and buy a bag of pasta made from lentils and peas, it costs five times as much as regular pasta, so if the other girl is having pasta too, we make regular pasta for her and lentil pasta for [EG], because otherwise … the cost of food increases too, so we have to make a lot of duplicate versions when we’re cooking …”* (Family E, father).

Another strategy enabling families to reduce costs was buying things online and in bulk. Some parents explained that while they spent more on some foods, they saved on others that were not permitted on the SCD and therefore no longer bought. Cooking simpler meals with fewer ingredients was also a strategy to reduce costs.

*“Depending on how you eat, more natural ingredients and stuff can be cheaper too, depending on how you do it. But it depends on how complicated … I don’t make complicated meals, like, so … that makes it cheaper.”* (family H, boy 20 years).

### Financial assistance

Most families noticed an increase in grocery costs after implementing the diet. Although they came up with various strategies to reduce their spending, they saw a risk that the cost could become a limiting factor for some families, restricting their access to dietary treatment. This financial limitation was considered unfair by many participants. To address the issue, a few parents suggested providing access to financial assistance similar to that offered to families in Sweden with a child with celiac disease which permits the purchase of gluten-free products at a subsidised price.

*“So I hope that the families that are maybe struggling can get help somehow. Like a subsidy, yeah, that you can get when it’s for medical reasons, somehow, so that you can get support.”* (Family M, mother).

### Dealing with socio-emotional consequences

For the children, the biggest challenges were socio-emotional. These were often the reason for returning to eating a regular diet again, despite having experienced symptom relief or anti-inflammatory effects. At home, things generally worked well, but many parents felt under-prepared for the challenges from the outside world. Some friends and family members questioned why they had decided to change their diet, and both children and parents felt left out in certain social situations. One family noticed how dinner invitations became infrequent after changing their diet. Most children had to take lunch to school, which made them feel different since everyone else ate the food served in the canteen. They found it burdensome to explain to their friends why they were bringing food from home. A couple of schools had policies preventing students from bringing food from outside the school into the canteen due to allergy risks. As a result of these policies, children had to heat their food elsewhere, such as in the teachers’ lounge, and eat alone. The children described lunch without their friends as ‘boring’ and they felt ‘unsocial’ and ‘excluded’. Being different was difficult. Another issue was a restricted social life. Being unable to do the same things as before led some to describe a feeling of being ‘stuck at home’. In the past, they could eat out at restaurants, go to a friend’s house after school without having to plan, or grab a snack from a store. They also struggled with feeling that it was unfair when friends and siblings could eat regular food, sweets and ice cream. This was especially hard during special occasions and holidays.

### Choosing to focus on the positive

One strategy used by both parents and children was to shift their focus away from dietary prohibitions and activities they struggled with, such as eating out with friends, and instead think of the SCD as an opportunity. Participants generally spoke about the diet as something positive and, apart from the potential health effects, it was regarded as an exciting opportunity to learn, try new foods, and contribute to research.

*“It’s good food, I would say, actually, because I feel like it’s good for wellbeing and health and so on, I would say that, actually. It would be good for everyone, really.”* (family M, mother).

The children also attempted to focus on the positive in particularly trying situations. After the first two weeks, many of the children became accustomed to the new diet and could appreciate previously unfamiliar tastes and enjoyed the new food cooked at home. One girl explained that most of her friends at school disliked the lunch from the canteen and were jealous of her because she was allowed to bring food from home. Despite not being allowed to eat in the canteen with her peers, another participant chose to highlight some positive outcomes of this, such as not being tempted by what others had on their plates and still being able to spend time with friends after finishing lunch. Another girl explained how she tried to deal with being faced with temptation:

*“Yeah, but I guess I thought that it’s better if I eat like this, because then it’ll be much better later.”* (Family B, girl 12 years).

### Supportive and encouraging environment

A supportive and encouraging environment was considered fundamental to managing the socio-emotional consequences, for both parents and children. Professional support was considered an essential aspect of the families’ ability to implement and adhere to the diet. Overall, the parents appreciated the support they had received during their time in the study. Dedication, praise and a positive attitude from research staff and medical professionals were all mentioned as things that helped give families the motivation to continue and made them feel at ease.

*“For one thing, we had a dietitian who was basically always there and we received contact information and so on, so we could contact them, and also we were there and talked to the doctor and then, and, yeah, no, and because we are there regularly anyway, you have a dialogue about it, so there was always support to make use of when we needed it, if we needed it.”* (Family E, father).

Within most families, the family members tried their best to support one another throughout the change. Even grandparents became involved and learned how to prepare SCD-friendly meals, lending much-appreciated support to the families. A few parents followed a strict diet to gain a better understanding of what their child was going through and be able to better support them during the intervention.

*“But we talked about it before we started, that everyone would be part of it, that we were doing this together. Then I felt that, yeah, I want to follow the exact same diet as FG, to understand what she’s going through and that we’re doing it together.”* (Family F, mother).

Children who had parents following the diet with them recognised this and appreciated their efforts.

*“My dad and my little brother, you know, they ate regular food, while my mum and I followed the diet, which made it much easier, when you don’t have to be the only person in the family eating it, so that was nice.”* (Family H, boy 20 years).

Parents recognised the importance of their child feeling like ‘a normal teenager’ and tried to preserve normalcy for them, to mitigate their feelings of being left out. However, this was easier at home than in social situations. One parent emphasised the value of not paying excessive attention to the diet when continuing it for a longer period, although to still retain a certain level of focus. They spent time, therefore, trying to come up with options that would be acceptable to the child and might resemble foods they enjoyed.

*“You tried to make everything as normal as possible. So, I mean, I tried to find dishes and things that could be a substitute so that he could also feel that he got tasty things and fancy things, like everyone else, and we adapted our diet too, we ate the same thing, so he wouldn’t feel like he was the odd one out at home.”* (Family A, mother).

### Preparation regarding socio-emotional aspects and communication

Before implementing the dietary change, most parents had a conversation with their other children, thoroughly explaining the situation and requesting their cooperation during the study. However, a few participants regretted not having had similar conversations with friends and family since they encountered confusion and resistance from some of them.

*“There was, like, a lot of misunderstanding about it. I think you have to work more with … like family and friends around you and prepare them too and ask them to respect what you are about to do.”* (Family G, mother).

There was a general awareness among parents regarding the awkwardness their children might feel in social situations, especially at school. It was perceived as likely to be most challenging for those in their early teenage years, referred to as a ‘sensitive age’. Parents suggested additional professional support from a social worker or psychologist to help them provide better support to their children in the face of unexpected psychosocial challenges. However, many parents were focused on the practical aspects and sometimes overlooked the emotional and psychological effects that the diet might have on their children.

*“One thing that maybe should be talked about a bit more when you’re doing this, is ‘So what do your friends at school say?’ and so on. If that’s something that he’s thought about. Because I think that he’s forgotten about it now and it wasn’t something we thought about then. We were just doing it and trying … like, to get it to work. So that is maybe something that you need to think about. How, like … yeah, how they deal with it. You just do what you have to do.”* (Family J, mother).

## Discussion

In this study we used an inductive qualitative approach to investigate how children with JIA and their parents experienced and navigated the challenges of the SCD, and to explore their needs for additional support. Parents and children were interviewed individually and together. Our results reflect experiences from a four-week intervention, as well as longitudinal experiences from families who continued to follow the diet after the study. The majority of the interviewed participants described it as an overall positive and beneficial experience despite having to face many challenges. For parents, these challenges tended to revolve around practical matters, whereas children struggled with the socio-emotional consequences.

One of the main strategies to deal with practical challenges was preparation and planning of food in order to create favourable conditions at home. Parents described how being prepared facilitated adherence to the diet in moments of stress or hunger. In line with this result, findings from a large population-based study investigating the association between meal planning and diet variety, quality and adherence showed that meal planning was associated with better adherence to dietary recommendations [24]. In our study, meal planning was not part of the intervention [11]. Nonetheless, in the interviews parents mentioned this as something they considered difficult – an essential skill they would like to learn. Although prior research has identified a strong interest among parents to learn meal planning [25–27], studies of interventions targeting food skills remain scarce [28].

Despite differences in both geographical location and participant diagnoses, most of our findings regarding the practical and economical aspects agreed with the results from the earlier qualitative study by Schwartz et al. [14]. However, our results were characterized by a strong emphasis on socio-emotional aspects. We believe the primary reason for this difference was the addition of the child perspective in our study. Unlike their parents, children found the socio-emotional consequences to be the most difficult to deal with. The increasing importance and influence of peers during adolescence, and their role in shaping food preferences and choices [29], may have contributed to these experiences. Another aspect to consider is the influence of the geographical and cultural context. For example, Sweden has a national school meal program, offering free lunch to all pupils. This draws attention to children with special diets and likely intensifies negative experiences, such as feelings of social exclusion already known to be an issue for children with dietary restrictions [30]. The letter from the principal investigator to the school kitchen staff, which was intended to inform them about a child’s participation in the study and request support with meal procedures, facilitated some of the practical aspects but failed to alleviate any negative experiences for the child related to the need to bring their own lunch. It is important to keep in mind that schools are not obligated to make accommodation for diets lacking proven scientific efficacy. Researchers should therefore remain vigilant regarding the participant’s situation at school, and establish and maintain open communication with both the school and the family to collaboratively identify ways to support a child on a special diet. This is particularly important in countries where lunch is provided at the school.

Another interesting finding is that the importance of socio-emotional consequences due to the dietary change was underestimated among some participants, which may indicate a potential risk of children’s struggles going unnoticed. One explanation for this may be that managing the practical aspects of a complex diet requires a high level of effort from parents, influencing their ability to give attention to the socio-emotional aspects being experienced by the child. Another reason for this finding may have been that children protect their parents by diluting or concealing their own negative feelings and struggles [31].

This study has some limitations. Firstly, since a small number of participants were interviewed four years after inclusion in the original study, recall bias cannot be excluded. However, the fact that many of the families continued the diet for several months or years after the study may have reduced the impact of this limitation. In addition, the presence of at least two family members during the interviews may have facilitated their ability to remember [32]. Secondly, we did not have the opportunity to interview participants who had discontinued the diet or decided not to participate. However, reasons for not initiating SCD previously described in the literature include multiple food allergies and low weight, and common reasons for discontinuation were lack of response and difficulty maintaining the diet [13, 14, 33, 34]. Finally, the mode of data collection (digital interviews) can be both a strength and a limitation. We considered online interviews suitable for the participants in this study, since the digital medium is a familiar method of communication for children and young adults. Additionally, it permitted them to be interviewed in their homes, possibly leading to richer data. Some also argue that digital interviews may increase the protection of privacy by providing a choice about whether or not to use a camera, and can also offer an additional level of control by enabling the participant to end the interview at any time [35]. This may also help counteract the inevitable power imbalance in interactions between children and adults. A disadvantage is that the quality of the recordings may be affected by internet connection or technical difficulties, which was the case in two of our interviews.

A strength of this study was including both child and parent perspectives, as this gave a more complete picture of the intervention experience. Dividing interviews into separate parts captured two perspectives of the same phenomenon, thus strengthening the trustworthiness of the results. In addition, this may have been helpful in minimizing, or at least indicating, social desirability bias based on discrepancy or congruency between the parents’ and children’s answers. Another strength was that the analysis was kept close to the material, minimising the risk of credibility loss due to overinterpretation [36].

### Future perspectives

The scope of this study was broad in order to explore the families’ experiences of this type of intervention. Our findings suggest multiple important aspects to consider in order to improve the experience for families. Future efforts could focus on ways to lessen the practical burden on parents by, for example, providing more quick, simple, and child-friendly recipes, as well as teaching parents practical, organisational food skills such as meal planning, and how to adapt their current meals to be SCD-compliant. The latter could also lessen the changes for the child, which may improve acceptability. Finally, the socio-emotional consequences of dietary treatment should not be underestimated and need to be given more attention. A question that remains to be answered is whether better preparing families to deal with both the practical and socio-emotional challenges, as well as providing professional socio-emotional support, could facilitate long-term adherence and improve the experience of dietary interventions such as the SCD.

## Conclusion

This study revealed that navigating treatment with the SCD was challenging in different ways, practically for parents and socio-emotionally for children. Based on the challenges reported and participants’ suggestions the intervention could be optimised by developing solutions to practical issues related to planning and food preparation, as well as better preparing families for dealing with the socio-emotional consequences of dietary change. In spite of the challenges and the need for additional support, the overall experiences and attitudes of the interviewed participants were positive to the current set-up. Consequently, dietary interventions, such as the SCD, may be regarded as suitable targets for further research.

### Electronic supplementary material

Below is the link to the electronic supplementary material.

**Additional File 1**. Semi-structured interview guide translated from Swedish to English

**Additional File 2**. Example of qualitative analysis using systematic text condensation

## Data Availability

The data supporting the findings of this study are not publicly available due to risk of participant identification. Data are located in controlled access data storage at Uppsala University.

## List of abbreviations

DMARDs: Disease-Modifying Antirheumatic Drugs
IBD: Inflammatory Bowel Disease
ILAR: International League of Associations for Rheumatology
JIA: Juvenile idiopathic arthritis
MTX: Methotrexate
RF: Rheumatoid factor
RF-: RF-negative polyarticular
RF+: RF-positive polyarticular
SCD: Specific Carbohydrate Diet
