# Supplementary material for: A qualitative evaluation of the specific carbohydrate diet for juvenile idiopathic arthritis based on children’s and parents’ experiences
Source: Pediatr Rheumatol Online J. 2023 Oct 19;21:127. doi: 10.1186/s12969-023-00914-8 (PMC10588234; doi:10.1186/s12969-023-00914-8)
Supplement: Supplementary file 1 — Additional File 1. Semi-structured interview guide translated from Swedish to English [file 12969_2023_914_MOESM1_ESM.docx]

| Additional File 1. Semi-structured interview guide translated from Swedish to English | |
| --- | --- |
| Area of interest | Questions |
| PART I – ONLY CHILD/YOUTH | |
| General experience | Can you tell me what it was like for you being part of the study?  Why did you want to take part?  What was the best thing about taking part? What was the worst thing?  What did you think of the diet or the food itself?  Is there any particular situation you remember while taking part/eating the food?  Can you tell me a bit about how it worked with lunch at school?  Did your friends know you were taking part in the study?   - How did they react? |
| Challenges | In what situation/when was it hardest for you to stick to the diet?   - Why was that?   How did you feel when others were eating something that you couldn’t eat? |
| Strategies/solutions | - What did you do/think in those situations? |
| Improvement | Can you give an example of something that could have helped you/made it easier to stick to the diet? |
| Final questions | Let’s say you could go back in time and your family was asked to participate in the study. Would you say yes, given what you know now after having taken part?   - Why is that?   Is there anything else you want to tell us or that you feel that we have missed? |
| PART II – COMMUNAL PART | |
| Expectations | What were your expectations before you started?   - Was there anything in particular that you thought would be easy/hard? - Did you think the diet would help with the disease?   Did it turn out the way you expected? |
| Past and current dietary habits | Had you ever tried a diet to treat the disease before?  If you think back to before you took part in the study, how did you usually eat then?  Do you eat together or at different times?  Does everyone eat the same thing or does that vary?   - Intolerances, special diets?   Did the entire family eat SCD (the same food) or only the child/youth?   - How long did you follow the diet?   How different would you say that the SCD was compared with how you ate before?   - What would you say was the biggest difference for you?   How does the family eat after the study?   - Have you gone back to eating like you did before or have you changed anything?   Have you gone back to the SCD?   - Would you consider doing so? |
| PART III – PARENT ONLY | |
| General question | Is there anything that we discussed in the communal part that you’d like to talk more about?  Can you tell me a bit about why you wanted to participate in the study and try this diet?  What do you think the child thought of participating in the study?   - What did their siblings think?   Can you tell me a bit more about how the diet worked in your everyday life? |
| Challenges | What challenges did you encounter?  Can you describe a situation where it was hard to follow the diet?  For some families, finances can be an obstacle to following this diet – what are your thoughts about that? |
| Strategies/solutions | In what ways did you need to adapt your routines to make things work in everyday life?   - Practical aspects/timewise?   How did you deal with situations where the child couldn’t eat the same things as their friends/family?  What advice would you give to other families that are about to start following SCD or participate in a similar study?  What advice would you like to give the researchers who performed the study? |
| Improvement | Can you give an example of anything that would have made it easier for your family to introduce or adhere to the dietary treatment?   - More/other type of support, information, or materials? |
| Final questions | Knowing what you know now, let’s say we can turn back the clock and your family is asked to participate in the study – would you say yes?   - Why is that?   Is there anything that you feel we have missed or that you want to talk more about? |
