# Supplementary material for: A qualitative evaluation of the specific carbohydrate diet for juvenile idiopathic arthritis based on children’s and parents’ experiences
Source: Pediatr Rheumatol Online J. 2023 Oct 19;21:127. doi: 10.1186/s12969-023-00914-8 (PMC10588234; doi:10.1186/s12969-023-00914-8)
Supplement: Supplementary file 2 — Additional File 2. Example of qualitative analysis using systematic text condensation [file 12969_2023_914_MOESM2_ESM.docx]

| Additional File 2. Example of qualitative analysis using systematic text condensation | | | |
| --- | --- | --- | --- |
| Step 1 | Step 2 | Step 3 | Step 4 |
| Preliminary theme:  Foods and cooking | Code group:  Doing what’s easiest in what’s difficult | Subgroup:  Simple, quick and child-friendly solutions | Category:  Managing practical issues |
| Transcripts | Meaning units | Condensed description | Final description |
| […] life will be much more … like, then you can have much more variety. *But I think that maybe simplifying what you already eat, trying to start from what the rest of the family is eating or … so you don’t have to work yourself ragged.* And then having a few, like, go-to’s… | But I think that maybe simplifying what you already eat, trying to start from what the rest of the family is eating or … so you don’t have to work yourself ragged. | To my mind it was a full-time job that it maybe didn’t have to be. It doesn’t have to be that hard if you start on a small scale, simplify what the family is already eating, so you don’t have to work yourself ragged. It’s easier to replace a product in something you already make than making something entirely new. I feel that it is really hard to find something that works for everyone in the family. The ambition is to not have lots of different meals. For us, it doesn’t work cooking two different dinners every day. They should eat what is served, but for picky kids the recipes maybe weren’t that great. There are a lot of strong spices in them and there maybe aren’t that many that kids like. It should be more regular recipes, simpler everyday food that kids and teenagers like. So we modified things ourselves, but then it became quite monotonous. In the end, she didn’t want to see bananas, pancakes or eggs, because that’s what was quick to make. Everything that was fast and easy, that worked for both the person eating and the person cooking. […] | The parents described managing everyday life while not making it too complicated while adhering to the diet as a ‘balancing act’. In hindsight, parents questioned the effort they had put into this, saying that perhaps they could have done things differently to make it simpler and less arduous. A common view was that cooking ‘shouldn’t take time; it needs to be quick’. Although most parents had the ambition that the whole family would eat the same food, several parents cooked multiple meals to please different family members. The recipes given elicited differing opinions among parents. Some found them helpful as meal suggestions and said they ‘would have been hard to manage without them’, while other families felt that quick and ‘simple everyday recipes that children and teenagers like’ were lacking. However, introducing new recipes was deemed to be too much of a change for some families. Many parents preferred to adapt their family recipes to work within the framework of the SCD |
| No, nothing. Nothing like that, no. Yeah, I’ve heard of it, but, like, yeah … *It’s too big a change with these recipe books. Or, yeah, the recipe sheets and … but it’s good to have them as a crutch. But then I changed most of the recipes. Yeah. Of course you can always do more, but that’s what worked for us.* Mm. The chicken soup, you liked that, so we still eat that. | It’s too big a change with these recipe books. Or, yeah, the recipe sheets and … but it’s good to have them as a crutch. But then I changed most of the recipes. Yeah. Of course you can always do more, but that’s what worked for us. |  |  |
